# Intranasal Netrin-1 modRNA-Loaded Extracellular Vesicles Reduce Neuronal Apoptosis after Ischemic Stroke

**DOI:** 10.34133/bmr.0423

**Published:** 2026-10-06

**Authors:** Shanshan Cao, Anmin Ren, Yanqin Geng, Hanlai Li, Yandi Qiao, Kangjie Fan, Yaohui Tang, Peng Wang, Qiang Li

**Affiliations:** ^1^ Department of Neurology, Gongli Hospital of Shanghai Pudong New Area, Shanghai 200135, China.; ^2^ Department of Cardiology, Shanghai General Hospital, Shanghai Jiao Tong University of Medicine, Shanghai 200080, China.; ^3^ School of Biomedical Engineering, Shanghai Jiao Tong University, Shanghai 200030, China.; ^4^ Tianjin Key Laboratory of Cerebral Vascular and Neurodegenerative Diseases, Tianjin Neurosurgical Institute, Tianjin Huanhu Hospital, Tianjin 300350, China.; ^5^ School of Gongli Hospital Medical Technology, University of Shanghai for Science and Technology, Shanghai 200093, China.; ^6^ Anti-Aging Center, Gongli Hospital of Shanghai Pudong New Area, Shanghai 200135, China.

## Abstract

Ischemic stroke remains a leading cause of mortality and long-term neurological disability worldwide, while effective neuroprotective therapies remain limited. Netrin-1 is a multifunctional guidance molecule with neuroprotective properties, and chemically modified mRNA (modRNA) offers a promising platform for transient therapeutic protein expression. However, safe and efficient delivery of modRNA to the ischemic brain remains a major barrier to clinical translation. Here, we engineered Netrin-1 modRNA-loaded extracellular vesicles (EV@mNetrin-1) derived from human umbilical cord mesenchymal stem cells (hUMSCs) and administered them via a noninvasive intranasal route. EV-mediated delivery enabled intracellular translation of Netrin-1 modRNA into protein in neurons and increased Netrin-1 expression in ischemic brain tissue. In vitro, EV@mNetrin-1 significantly reduced neuronal apoptosis and improved neuronal survival following oxygen–glucose deprivation/reoxygenation (OGD/R). In vivo, intranasal administration of EV@mNetrin-1 enhanced brain Netrin-1 expression, reduced infarct volume, improved neurological outcomes, and attenuated neuronal apoptosis in transient middle cerebral artery occlusion (tMCAO) mice. Mechanistically, the neuroprotective effects of EV@mNetrin-1 were associated with DCC-dependent activation of PI3K/AKT signaling. Collectively, these findings demonstrate the therapeutic potential of intranasal EV-mediated Netrin-1 modRNA delivery for ischemic stroke and support the development of RNA therapeutics for central nervous system disorders.

## Introduction

Stroke remains one of the leading causes of mortality and long-term disability worldwide, imposing a substantial socioeconomic burden on healthcare systems. Ischemic stroke accounts for approximately 80% of all stroke cases. Although reperfusion therapies, including intravenous thrombolysis and mechanical thrombectomy, have markedly improved clinical outcomes, their applicability remains limited by narrow therapeutic windows and strict eligibility criteria [1]. Moreover, reperfusion itself may exacerbate neuronal injury through secondary injury cascades, further compromising neurological recovery [2]. Neuronal apoptosis is one of the major contributors to ischemic brain injury. However, despite decades of investigation, most neuroprotective strategies have failed to achieve clinical translation, largely owing to inefficient delivery across the blood–brain barrier (BBB) and insufficient target engagement in the ischemic brain [3]. Therefore, developing an efficient and clinically applicable delivery strategy for neuroprotective agents remains a major challenge in ischemic stroke therapy.

Netrin-1 is a secreted laminin-related protein with diverse biological functions and has been shown to exert neuroprotective effects in experimental stroke models, including modulation of inflammation, preservation of BBB integrity, and inhibition of neuronal apoptosis [4]. These protective effects are mediated through receptor-dependent signaling. Netrin-1 interacts with multiple receptors, including deleted in colorectal cancer (DCC), neogenin, and members of the UNC5 receptor family. Netrin-1/DCC signaling activates downstream prosurvival pathways and suppresses ischemia-induced neuronal apoptosis [4]. Previous studies have demonstrated that intracerebral administration of recombinant Netrin-1 protein or viral vector-mediated Netrin-1 overexpression improves neurological function in experimental ischemic stroke models [5,6]. In addition, elevated circulating Netrin-1 levels have been associated with favorable clinical outcomes in patients with ischemic stroke [7]. However, the therapeutic application of Netrin-1 is constrained by the lack of an efficient delivery strategy capable of achieving sustained protein expression within the ischemic brain.

To address the limitations of conventional protein therapy, chemically modified messenger RNA (modRNA) has emerged as a versatile platform for transient protein expression with rapid onset and low immunogenicity, offering a promising strategy for in situ production of therapeutic proteins [8]. Nevertheless, naked modRNA is prone to rapid degradation and exhibits poor cellular uptake, necessitating the development of effective delivery systems. Multiple delivery platforms, including lipid nanoparticles (LNPs) and extracellular vesicles (EVs), are currently under intensive investigation. Although LNPs have revolutionized mRNA vaccine development, their application in central nervous system diseases remains limited by potential immunogenicity and toxicity, preferential hepatic accumulation, and suboptimal tissue-specific delivery [9,10]. In contrast, EVs, particularly those derived from human umbilical cord mesenchymal stem cells (hUMSCs), have emerged as promising natural nucleic acid carriers owing to their favorable biocompatibility, low immunogenicity, capacity to deliver bioactive cargo, and inherent therapeutic potential [11–13]. Moreover, EVs can be engineered to load exogenous nucleic acids, such as modRNA, while preserving the intrinsic therapeutic properties inherited from their parental cells, thereby providing dual therapeutic benefits [10,14]. Recent studies have demonstrated the therapeutic potential of engineered EV-mediated modRNA delivery in tissue repair. For example, engineered exosomes enriched with Netrin-1 modRNA have been shown to promote spinal cord repair, while M2 macrophage-derived exosomes carrying modRNA encoding nerve growth factor (NGF) facilitate corneal regeneration, highlighting the versatility of this delivery platform [15,16].

Nevertheless, achieving efficient and noninvasive delivery of nucleic acid therapeutics to the brain remains a major challenge. Intranasal administration has emerged as an attractive strategy for central nervous system drug delivery because it enables therapeutic agents to bypass the BBB via olfactory and trigeminal pathways [17]. Previous studies have demonstrated that intranasally administered hUMSC-derived EVs can be internalized by neural cells in the ischemic brain region of transient middle cerebral artery occlusion (tMCAO) mice [18]. Collectively, these findings suggest that the combination of engineered EVs with intranasal administration provides a promising strategy for targeted brain delivery of nucleic acid therapeutics. Despite these advances, engineered EV-mediated modRNA delivery has shown therapeutic potential in other disease models; however, its application in ischemic stroke remains largely unexplored. To our knowledge, no study has established a noninvasive platform for intranasal delivery of Netrin-1 modRNA using hUMSC-derived EVs that simultaneously enables sustained Netrin-1 expression and harnesses the intrinsic therapeutic properties of EVs.

Guided by this rationale, we established an intranasal EV-mediated modRNA delivery platform by loading chemically modified Netrin-1 mRNA into hUMSC-derived EVs (EV@mNetrin-1). We investigated whether this noninvasive EV-based modRNA delivery strategy could enhance Netrin-1 expression within the ischemic brain, attenuate neuronal apoptosis, reduce infarct volume, and improve neurological function. Furthermore, we explored the involvement of the DCC signaling pathway in mediating the neuroprotective effects of EV@mNetrin-1. Beyond providing a promising noninvasive therapeutic strategy for ischemic stroke, this study may provide a foundation for the development of EV-mediated modRNA delivery for neurological disorders.

## Materials and Methods

### Isolation and characterization of hUMSC-derived EVs

hUMSCs (Procell, China) were cultured in Dulbecco’s modified Eagle’s medium (DMEM)/F12 medium (Gibco, USA) supplemented with 10% fetal bovine serum (FBS). When cells reached approximately 80% confluence, the culture medium was replaced with DMEM/F12 containing 10% EV-depleted FBS for an additional 48 h. EV-depleted FBS was prepared by ultracentrifugation at 100,000*g* overnight at 4 °C. Conditioned medium was subsequently collected and subjected to sequential differential centrifugation to remove cells and cellular debris. Briefly, the supernatant was centrifuged at 2,000*g* for 20 min, followed by 10,000*g* for 30 min at 4 °C. The resulting supernatant was filtered through a 0.22-μm membrane filter to remove larger vesicles and apoptotic bodies. EVs were then isolated by ultracentrifugation at 100,000*g* for 70 min at 4°C. The EV pellet was washed once with sterile phosphate-buffered saline (PBS) and ultracentrifuged again at 100,000*g* for 70 min to further improve purity. Finally, purified EVs were resuspended in sterile PBS and stored at −80 °C for subsequent experiments.

For EV characterization, EV morphology was examined by transmission electron microscopy (TEM; Tecnai G2 Spirit BioTWIN, Thermo Fisher Scientific, USA). Particle size distribution and particle concentration were analyzed using nanoparticle tracking analysis (NTA; ZetaView, Particle Metrix GmbH, Germany). Total EV protein content was quantified using a BCA Protein Assay Kit (Meilunbio, China) according to the manufacturer’s instructions. Expression of EV markers, including CD9, CD63, CD81, and TSG101, as well as the negative EV marker calnexin, was evaluated by Western blotting.

### Synthesis of modRNA and EV loading

modRNAs encoding Netrin-1, enhanced green fluorescent protein (EGFP), or luciferase were synthesized by in vitro transcription as previously described [19]. The synthesized modRNAs were purified and quantified before use. For EV loading, modRNAs were incorporated into EVs using an Exo-Fect Exosome Transfection Kit (System Biosciences, USA) according to the manufacturer’s instructions with minor modifications. Briefly, 100 μl of purified EVs (1 × 10^10^ particles) were mixed with modRNA solution, PBS, and 10 μl of Exo-Fect reagent to a final volume of 150 μl. For in vitro experiments, 10 μl of modRNA solution was used, whereas 15 μl of modRNA solution was used for in vivo experiments. The mixture was incubated at 37 °C for 10 min under gentle agitation. Subsequently, 30 μl of ExoQuick-TC reagent was added, followed by incubation on ice for 30 min. The modRNA-loaded EVs were collected by centrifugation at 15,000 rpm for 3 min. The supernatant containing unencapsulated modRNA and residual loading reagents was discarded, and the EV pellet was resuspended in PBS for subsequent experiments.

### Evaluation of modRNA encapsulation efficiency

The encapsulation efficiency (EE) of Netrin-1 modRNA was determined using a RiboGreen RNA Assay Kit (Solarbio, Beijing, China), as previously described by Gong et al. [16]. Briefly, EV@mNetrin-1 samples were treated with 0.5% (w/v) Triton X-100 to disrupt EV membranes and determine total RNA, whereas untreated EV@mNetrin-1 samples were used to quantify free (unencapsulated) RNA. Naïve EVs were processed in parallel to account for interference from endogenous EV-associated RNA. Fluorescence was measured at excitation/emission wavelengths of 485/538 nm. EE was calculated as follows:

$$\mathrm{EE}\left(\%\right)=\frac{{\mathrm{RNA}}_{\text{total}}-{\mathrm{RNA}}_{\text{free}}-{\mathrm{RNA}}_{\text{endo}}}{{\mathrm{RNA}}_{\text{total}}-{\mathrm{RNA}}_{\text{free}}}\times 100$$ (1)

where ${\mathrm{RNA}}_{\text{total}}$, ${\mathrm{RNA}}_{\text{free}}$, and ${\mathrm{RNA}}_{\text{endo}}$ represent the RNA concentrations measured in Triton X-100-treated EV@mNetrin-1, untreated EV@mNetrin-1, and Triton X-100-treated naïve EVs, respectively.

### EV labeling with PKH26 and DiR

EVs were labeled with PKH26 or DiR fluorescent dyes for cellular uptake and biodistribution studies, respectively. For PKH26 labeling, EVs were stained using a PKH26 Red Fluorescent Cell Linker Kit (Umibio, Shanghai, China) according to the manufacturer’s instructions. PKH26-labeled EVs were added to the cell culture medium for in vitro uptake assays or administered intranasally to mice for immunofluorescence analysis. For DiR labeling, EVs were incubated with DiR dye (Meilun Biotechnology, Dalian, China) at a final concentration of 10 μM for 30 min at 37 °C under gentle agitation. Subsequently, PBS was added to dilute the EV–dye mixture, followed by ultracentrifugation at 100,000*g* for 70 min at 4 °C to remove unbound dye. The labeled EV pellet was resuspended in PBS for subsequent experiments.

### Animals

Male C57BL/6J mice (8 to 10 weeks old, 22 to 25 g) were purchased from GemPharmatech Co. Ltd. (Nanjing, China) and housed under specific pathogen-free (SPF) conditions with a 12-h light/dark cycle at 22 °C and 50% to 60% humidity, with free access to food and water. All animal experiments were approved by the Institutional Animal Care and Use Committee (IACUC) of Shanghai Jiao Tong University, Shanghai, China, and were performed in accordance with institutional guidelines for animal care and use.

### The mouse model of tMCAO

tMCAO was performed as previously described [20]. Mice were anesthetized with 4% isoflurane and maintained at 1.5% isoflurane throughout the procedure. Following a midline cervical incision, the common carotid artery (CCA), external carotid artery (ECA), and internal carotid artery (ICA) were carefully exposed. A silicone-coated nylon filament (Jialing Biotechnology, Guangzhou, China) was inserted into the ICA through the ECA stump to occlude the origin of the middle cerebral artery (MCA). After 90 min of occlusion, the filament was withdrawn to allow reperfusion. Regional cerebral blood flow (rCBF) in the MCA territory was continuously monitored using laser Doppler flowmetry (Moor Instruments, UK). Successful occlusion was defined as a reduction in rCBF to <20% of baseline during occlusion, followed by recovery to >80% upon reperfusion. Animals that did not meet these criteria were excluded from further analysis. In addition, mice with evidence of subarachnoid hemorrhage during surgery or tissue collection were excluded. Mice were randomly assigned to the PBS, EV, and EV@mNetrin-1 groups after tMCAO. Sham-operated mice underwent the same surgical procedures without insertion of the filament into the ICA.

### Intranasal administration

Intranasal delivery was carried out as previously described [21]. To enhance nasal permeability, hyaluronidase (100 U in 5 μl per nostril) was administered 30 min prior to treatment. Mice were then intranasally treated with EV or EV@mNetrin-1 at a dose of 1.0 × 10^10^ particles per mouse. The same volume of PBS was administered to the control group, while sham-operated mice received no treatment. All administrations were performed 3 h after tMCAO induction.

### In vivo and ex vivo fluorescence imaging

DiR-labeled EVs were used for in vivo and ex vivo fluorescence imaging. At 3, 6, 12, and 24 h after intranasal administration, mice were anesthetized with isoflurane (4% for induction and 1.5% for maintenance), and the cranial region was shaved prior to imaging. Near-infrared fluorescence (NIRF) images were acquired using an IVIS Spectrum imaging system (PerkinElmer, MA, USA). For ex vivo imaging, mice were euthanized at 24 h after administration, and major organs, including the brain, heart, lungs, liver, kidneys, and spleen, were harvested for fluorescence imaging. Fluorescence images were acquired with excitation and emission wavelengths of 750 and 782 nm, respectively. Fluorescence signals were quantified using Living Image software and expressed as average radiant efficiency.

### Neurobehavioral assessments

Neurological function was evaluated at 3 d after intranasal administration by an investigator blinded to group allocation. The modified neurological severity score (mNSS) was used to evaluate motor and balance functions. Scores ranged from 0 to 12, with higher scores indicating more severe neurological deficits. Motor asymmetry was further assessed using the elevated body swing test (EBST). Briefly, mice were suspended by the tail, and the direction of each body swing was recorded over 20 consecutive trials. Results were expressed as the percentage of right-biased swings relative to the total number of trials.

### Assessment of infarct volume

Cerebral infarct volume was assessed at 3 d after tMCAO. Mice were transcardially perfused with PBS followed by 4% paraformaldehyde (PFA), and brains were harvested and frozen at −80 °C. Serial coronal sections (20 μm thickness) were collected at 200-μm intervals and stained with 0.1% cresyl violet (Meilun Biotechnology, Dalian, China). Infarct areas were quantified using ImageJ software. To correct for brain edema, the infarct area in each section was calculated as the area of the contralateral hemisphere minus the non-infarcted area of the ipsilateral hemisphere. Infarct volume was estimated using the following formula: *V* = ∑ *h*/3 × [Δ*S_n_* + (Δ*S_n_* × Δ*S*_*n*+1)_^1/2^ + Δ*S*_*n*+1_], where *V* represents the infarct volume, *h* is the distance between adjacent sections (200 μm), and Δ*S_n_* and Δ*S*_*n*+1_ denote the infarct areas of 2 consecutive sections after edema correction.

### Primary neuron culture and OGD/R model

Primary cortical neurons were isolated from neonatal C57BL/6 mice. Briefly, cerebral cortices were rapidly dissected, mechanically dissociated, and digested with 0.05% trypsin (Gibco, USA). Digestion was terminated by adding DMEM containing 10% FBS. The cell suspension was filtered through a 40-μm cell strainer and centrifuged to obtain a single-cell suspension. Cells were seeded onto poly-d-lysine-coated culture plates (Sigma-Aldrich, USA) and maintained in DMEM for initial attachment. After 2 h, the medium was replaced with Neurobasal-A medium (Gibco, USA) supplemented with B-27 (Gibco, USA). Neurons were cultured at 37 °C in a humidified incubator containing 5% CO₂, and half of the medium was replaced every 3 d. Neurons at 7 to 10 d in vitro were used for subsequent experiments.

To establish an in vitro ischemia/reperfusion model, oxygen–glucose deprivation/reoxygenation (OGD/R) was performed. Briefly, neurons were transferred to glucose-free DMEM pre-equilibrated under hypoxic conditions and incubated in an anaerobic chamber containing 95% N₂ and 5% CO₂ for 8 h. After OGD, cells were returned to normoxic conditions and cultured in Neurobasal-A medium for reoxygenation. Neurons were subsequently treated with EVs loaded with Netrin-1 modRNA (EV@mNetrin-1), naïve EVs (EVs), naked Netrin-1 modRNA (mNetrin-1), EVs loaded with luciferase modRNA (EV@mLuc), or PBS and were collected for subsequent analyses after 24 h.

### Evaluation of EV-mediated modRNA expression in primary neurons

To evaluate EV-mediated modRNA expression in neurons, primary cortical neurons were seeded in 24-well plates and treated with EV@mEGFP at a dose of 1 × 10^9^ particles per well. The same wells were longitudinally monitored for EGFP fluorescence at 12, 24, 48, 72, 96, 120, and 144 h after treatment. Fluorescence images were acquired using identical imaging settings across all time points. EGFP fluorescence intensity was quantified using ImageJ software and normalized to the corresponding fluorescence intensity at 12 h.

### Cell viability assay

Cell viability was assessed under both normal culture and OGD/R conditions. To evaluate cytocompatibility under normal culture conditions, primary neurons were treated with mNetrin-1, EV, EV@mLuc, or EV@mNetrin-1 for 24 h, with untreated neurons serving as the control. To evaluate the neuroprotective effects after OGD/R, primary neurons were treated with EV@mNetrin-1, EV, mNetrin-1, EV@mLuc, or PBS immediately after reoxygenation and cultured under normal conditions for 24 h. Cell viability was assessed using a Cell Counting Kit-8 (CCK-8; Meilun Biotechnology, Dalian, China) according to the manufacturer’s instructions. Briefly, CCK-8 reagent was added to each well and incubated at 37 °C for 1 h. Absorbance at 450 nm was measured using a microplate reader (BioTek, USA).

### TUNEL staining assay

Cells and brain sections were fixed with 4% PFA. Apoptotic cells were detected using a terminal deoxynucleotidyl transferase–mediated deoxyuridine triphosphate nick end labeling (TUNEL) assay kit (Meilun Biotechnology, Dalian, China) according to the manufacturer’s instructions. Nuclei were counterstained with 4′,6-diamidino-2-phenylindole (DAPI), and images were acquired using a fluorescence microscope. For quantitative analysis, 3 randomly selected fields per sample were analyzed. In cultured neurons, apoptosis was quantified as the percentage of TUNEL-positive cells among total DAPI-positive cells. In brain sections, neuronal apoptosis was quantified as the percentage of TUNEL-positive cells among NeuN-positive neurons.

### Immunofluorescence staining

Immunofluorescence staining was performed on cultured cells and brain tissue sections. Samples were fixed with 4% PFA, permeabilized with 0.3% Triton X-100 for 10 min, and blocked with 1% bovine serum albumin for 1 h at room temperature. Samples were then incubated with primary antibodies overnight at 4 °C. The following primary antibodies were used: Iba1 (1:200; WAKO, Japan), glial fibrillary acidic protein (GFAP) (1:400; Abcam, UK), NeuN (1:300; Abcam, UK), MAP2 (1:250; Proteintech, China), and Netrin-1 (1:200; Proteintech, China). After washing with PBS, samples were incubated with Alexa Fluor-conjugated secondary antibodies (1:500; Invitrogen, USA) for 1 h at room temperature. Nuclei were counterstained with DAPI (1:2,000; Beyotime, China). Fluorescence images were acquired using a confocal laser scanning microscope or fluorescence microscope (Leica, Germany).

### siRNA-mediated DCC knockdown

DCC knockdown was performed using small interfering RNA (siRNA). A nontargeting scrambled siRNA was used as the negative control. Primary cortical neurons at 5 to 7 d were transfected with DCC-specific siRNA (final concentration: 100 nM; SYNBIO Technologies, Suzhou, China) using Lipofectamine RNAiMAX (Invitrogen, USA) according to the manufacturer’s instructions. Briefly, siRNA and Lipofectamine RNAiMAX were separately diluted in Opti-MEM (Gibco, USA). The diluted solutions were gently mixed and incubated for 15 min at room temperature to allow complex formation before being added to the cells. Total RNA was harvested at 24 h after transfection for quantitative polymerase chain reaction (qPCR) analysis, while protein samples were collected at 48 h for Western blot analysis to evaluate knockdown efficiency. Subsequent experiments were initiated 48 h after transfection, when DCC protein knockdown was pronounced. The sequences targeting DCC were as follows: sense, 5′-GGAAAUGAAGCAGAAGUUAGATT-3′ and antisense, 5′-UCUAACUUCUGCUUCAUUUCCTT-3′. The scrambled siRNA sequences were sense, 5′-UUCUCCGAACGUGUCACGUdTdT-3′ and antisense, 5′-ACGUGACACGUUCGGAGAAdTdT-3′.

### Quantitative real-time PCR

Total RNA from EV samples was isolated using an Exosome RNA Isolation Kit (Umibio, Shanghai, China; UR31013) according to the manufacturer’s instructions. Total RNA from cultured cells and ischemic brain tissues was extracted using TRIzol reagent (Invitrogen, USA). RNA concentration and purity were determined using a NanoDrop spectrophotometer (Thermo Fisher Scientific, USA). cDNA was synthesized using HiScript II Q RT SuperMix for qPCR (+gDNA wiper) (Vazyme, Nanjing, China) according to the manufacturer’s instructions. Quantitative real-time PCR was performed using ChamQ Blue Universal SYBR qPCR Master Mix (Vazyme, China) on a 7900HT Real-Time PCR System (Applied Biosystems, USA) under the following conditions: 95 °C for 30 s, followed by 40 cycles of 95 °C for 5 s and 60 °C for 30 s. Gene expression levels were normalized to β-actin and analyzed using the 2^−ΔΔ*C*t^ method. Relative mRNA levels were expressed as fold changes compared with the corresponding control group. The primer sequences used in this study are listed in Table S1.

### Western blot analysis

Protein expression levels were analyzed by Western blotting. Total proteins were extracted from EVs, cultured cells, and ischemic brain tissues using radioimmunoprecipitation assay (RIPA) lysis buffer (Millipore, USA) supplemented with protease and phosphatase inhibitors. Protein concentrations were determined using a BCA Protein Assay Kit according to the manufacturer’s instructions. Equal amounts of total protein were separated by sodium dodecyl sulfate–polyacrylamide gel electrophoresis (SDS-PAGE) and transferred onto polyvinylidene difluoride (PVDF) membranes. Membranes were blocked and incubated overnight at 4 °C with the following primary antibodies: CD81 (Beyotime, China), TSG101 (Selleck Chemicals, China), calnexin (Selleck Chemicals, China), CD9 (Cell Signaling Technology, USA), CD63 (Proteintech, China), Netrin-1 (Proteintech, China), DCC (HUABIO, China), BCL2 (Proteintech, China), BAX (Proteintech, China), cleaved caspase-3 (Proteintech, China), phosphatidylinositol 3-kinase (PI3K) (HUABIO, China), phosphorylated PI3K (p-PI3K; HUABIO, China), AKT (HUABIO, China), phosphorylated AKT (p-AKT; HUABIO, China), and β-actin (Proteintech, China). After washing, membranes were incubated with appropriate horseradish peroxidase (HRP)-conjugated secondary antibodies (Proteintech, China) for 1 h at room temperature. Protein bands were visualized using enhanced chemiluminescence (ECL) reagents. Band intensities were quantified using ImageJ software. For cellular and tissue samples, target protein levels were normalized to β-actin and expressed as fold changes relative to the control group. The p-PI3K/PI3K, p-AKT/AKT, and BCL2/BAX ratios were calculated based on densitometric analysis of the corresponding protein bands.

### Statistical analysis

Statistical analyses were conducted using GraphPad Prism software (version 10.0; GraphPad Software, San Diego, CA, USA). Data are presented as mean ± standard deviation (SD). Behavioral assessments were performed by investigators blinded to group allocation. Comparisons between 2 groups were performed using an unpaired 2-tailed Student’s *t* test. Comparisons among multiple groups were analyzed using one-way analysis of variance (ANOVA) followed by Tukey’s multiple-comparisons test. For ex vivo biodistribution analysis, comparisons between the sham and EV-DiR groups for each organ were performed using multiple unpaired 2-tailed Welch’s *t* tests with the Holm–Šídák correction for multiple comparisons. *P* < 0.05 was considered statistically significant.

## Results

### EV-mediated modRNA delivery enables efficient neuronal expression

To establish an EV-based modRNA delivery platform, we engineered hUMSC-derived EVs loaded with Netrin-1 modRNA (EV@mNetrin-1). EVs were isolated from hUMSCs and subsequently loaded with Netrin-1 modRNA using the Exo-Fect reagent (Fig. 1A).

**Fig. 1.**
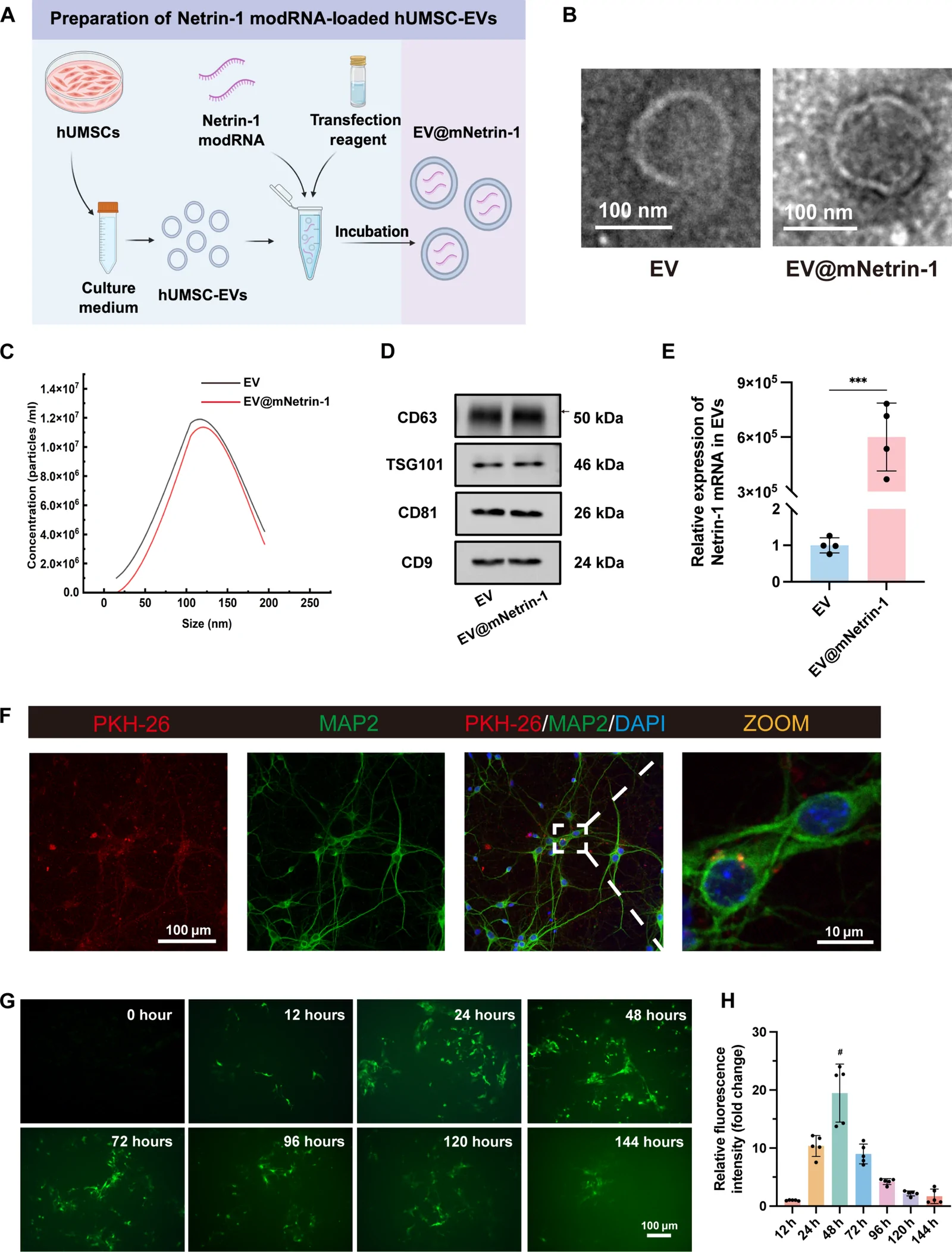
Construction and characterization of an EV-based modRNA delivery system. (A) Schematic illustration of EV@mNetrin-1 construction through Exo-Fect-mediated loading of Netrin-1 modRNA into hUMSC-derived EVs. (B) TEM images showing the characteristic cup-shaped morphology of EVs and EV@mNetrin-1. (C) NTA showing the size distributions of EVs and EV@mNetrin-1. (D) Western blot analysis of canonical EV-associated markers (CD9, CD63, CD81, and TSG101) in EVs and EV@mNetrin-1. (E) RT-qPCR analysis of Netrin-1 mRNA levels in EV and EV@mNetrin-1. (F) Immunofluorescence analysis of PKH26-labeled EV uptake by MAP2-positive primary neurons after 6 h of incubation. Scale bars, 100 μm or 10 μm. (G) Representative EGFP fluorescence images of primary neurons following treatment with EV@mEGFP at the indicated time points. Scale bars, 100 μm. (H) Quantitative analysis of EGFP fluorescence intensity at the indicated time points following EV@mEGFP treatment. Fluorescence intensity was normalized to the 12-h time point. Data are presented as mean ± SD. ***P < 0.001 in (E); ^#^*P* < 0.05 for the 48-h time point versus other indicated time points in (H).

TEM demonstrated the characteristic cup-shaped morphology of EVs and EV@mNetrin-1 (Fig. 1B). NTA showed that both EVs and EV@mNetrin-1 exhibited nanoscale size distributions, with modal diameters of 124.4 nm and 140.1 nm, respectively (Fig. 1C and Fig. S1A). Canonical EV-associated markers (CD63, TSG101, CD81, and CD9) were detected, whereas calnexin was absent (Fig. 1D and Fig. S1C), confirming successful EV characterization and preservation of EV properties after modRNA loading.

We next evaluated the loading of Netrin-1 modRNA into EVs. The EE of Netrin-1 modRNA was 52.1% (Fig. S1D). Real-time qPCR (RT-qPCR) further confirmed successful loading, with Netrin-1 mRNA levels in EV@mNetrin-1 being approximately 6 × 10^5^-fold higher than those in EVs (Fig. 1E).

To assess whether EVs could be internalized by neurons, EVs were labeled with PKH26 and incubated with primary neurons. Immunofluorescence staining demonstrated that PKH26-labeled EVs were efficiently taken up by MAP2-positive neurons after 6 h of incubation (Fig. 1F).

We then determined whether EV-delivered modRNA could be translated into protein in neurons. EVs loaded with EGFP modRNA (EV@mEGFP) were generated as a reporter system. Primary neurons treated with EV@mEGFP exhibited a dynamic EGFP expression profile, with fluorescence detectable at 12 h, reaching the highest measured intensity at 48 h, and gradually declining thereafter while remaining detectable up to 144 h (Fig. 1G and H).

Taken together, these results demonstrate that engineered EVs can efficiently encapsulate and deliver modRNA into neurons, enabling robust and sustained protein expression.

### EV@mNetrin-1 enhances neuronal Netrin-1 expression and reduces apoptosis after OGD/R

To determine whether EV@mNetrin-1 enhances neuronal Netrin-1 expression, primary neurons were treated with mNetrin-1 (naked Netrin-1 modRNA), EV, EV@mLuc (EV loaded with luciferase modRNA), or EV@mNetrin-1 under normal culture conditions, with untreated neurons serving as the control. Western blot analysis demonstrated significantly increased Netrin-1 protein expression in the EV@mNetrin-1 group compared with all other groups (Fig. 2A and B). We also assessed the cytocompatibility of EV@mNetrin-1 under normal culture conditions. No significant differences in neuronal viability were observed among the groups by CCK-8 assay (Fig. 2C), indicating no evident cytotoxicity of EV@mNetrin-1.

**Fig. 2.**
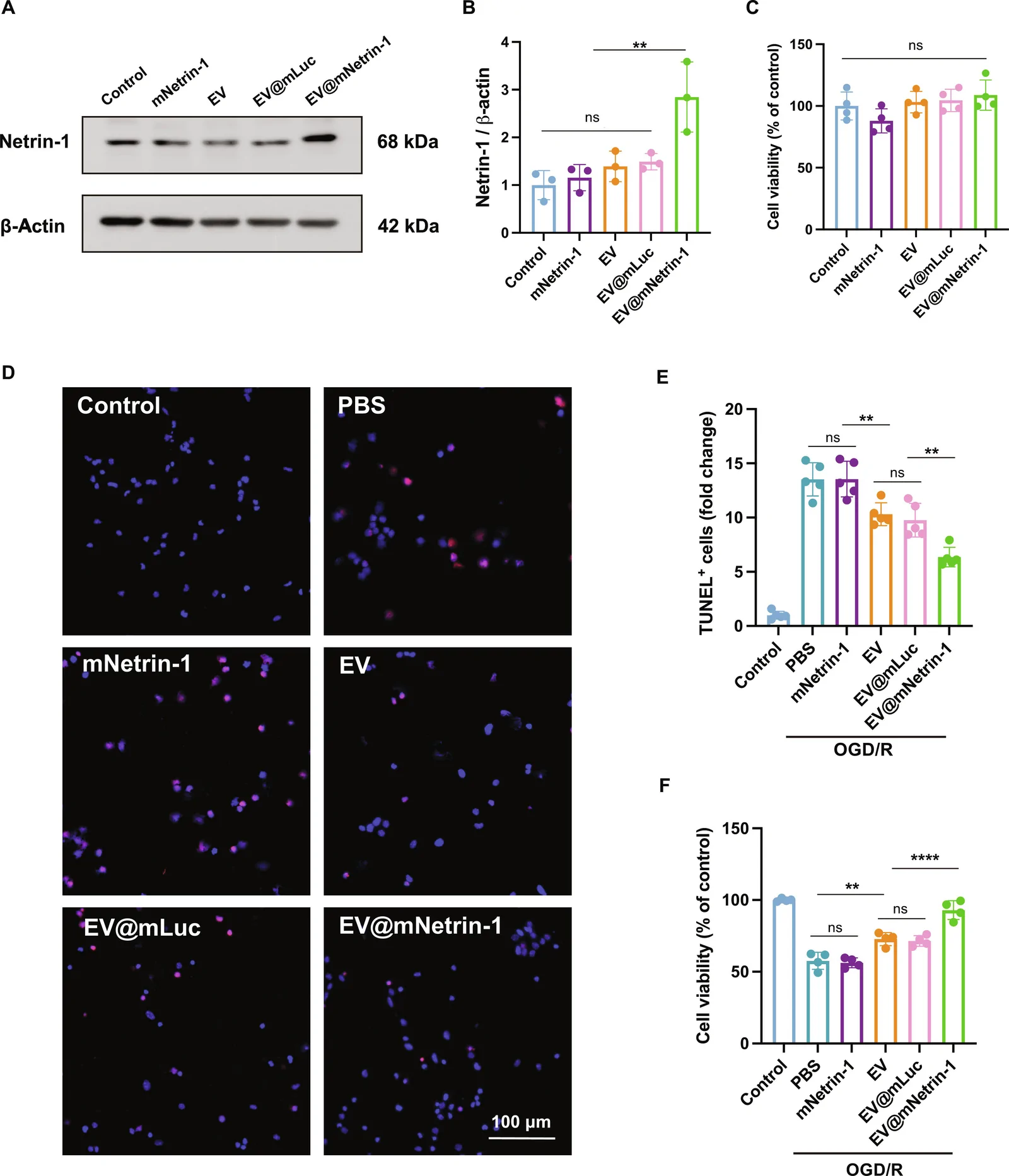
EV@mNetrin-1 enhances neuronal Netrin-1 expression and protects neurons against OGD/R-induced injury. (A) Western blot analysis of Netrin-1 protein expression in primary neurons under normal culture conditions following 24-h treatment with mNetrin-1, EV, EV@mLuc, or EV@mNetrin-1, with untreated neurons as the control. *N* = 3 per group. (B) Quantification of Netrin-1 protein expression shown in (A). (C) CCK-8 assay of neuronal viability under normal culture conditions. *N* = 4 per group. (D) Representative images of TUNEL staining in primary neurons subjected to OGD/R and treated with PBS, mNetrin-1, EV, EV@mLuc, or EV@mNetrin-1. *N* = 5 per group. Scale bars, 100 μm. (E) Quantification of TUNEL-positive cells shown in (D). (F) CCK-8 assay of neuronal viability following OGD/R. *N* = 4 per group. Data are presented as mean ± SD. *P < 0.05; **P < 0.01; ****P < 0.0001; ns, not significant. EV@mNetrin-1, EVs loaded with Netrin-1 modRNA; EV@mLuc, EVs loaded with luciferase modRNA; mNetrin-1, naked Netrin-1 modRNA.

We next investigated whether EV@mNetrin-1 could exert neuroprotective effects under ischemic conditions. Neurons were subjected to OGD/R and subsequently treated with PBS, mNetrin-1, EV, EV@mLuc, or EV@mNetrin-1, with an additional non-OGD control group. TUNEL staining showed that neuronal apoptosis was reduced in the EV, EV@mLuc, and EV@mNetrin-1 groups compared with the PBS group (Fig. 2D and E). Among these groups, EV@mNetrin-1 treatment further decreased neuronal apoptosis compared with EV and EV@mLuc treatment. No significant differences were observed between the EV and EV@mLuc groups or between the PBS and mNetrin-1 groups.

Consistent with the TUNEL results, CCK-8 assay demonstrated increased neuronal viability in the EV, EV@mLuc, and EV@mNetrin-1 groups after OGD/R compared with the PBS group (Fig. 2F). Moreover, EV@mNetrin-1 treatment further enhanced neuronal viability relative to EV and EV@mLuc treatment, whereas no significant differences were detected between the EV and EV@mLuc groups or between the PBS and mNetrin-1 groups.

Taken together, these results demonstrate that EV@mNetrin-1 enhances neuronal Netrin-1 expression and protects neurons against OGD/R-induced injury.

### Intranasal administration enables EV delivery to the ischemic brain and uptake by neural cells

To determine whether intranasally administered EVs could reach the brain, DiR-labeled EVs were administered intranasally and monitored by in vivo imaging at 3, 6, 12, and 24 h post-administration, with PBS-treated mice serving as the sham control. In vivo imaging demonstrated a time-dependent increase in fluorescence signal in the brains of EV-treated mice (Fig. 3A). Quantitative analysis of average radiant efficiency showed that the fluorescence signal was significantly increased as early as 3 h, peaked at 12 h, and remained elevated at 24 h compared with the sham group (Fig. 3B).

**Fig. 3.**
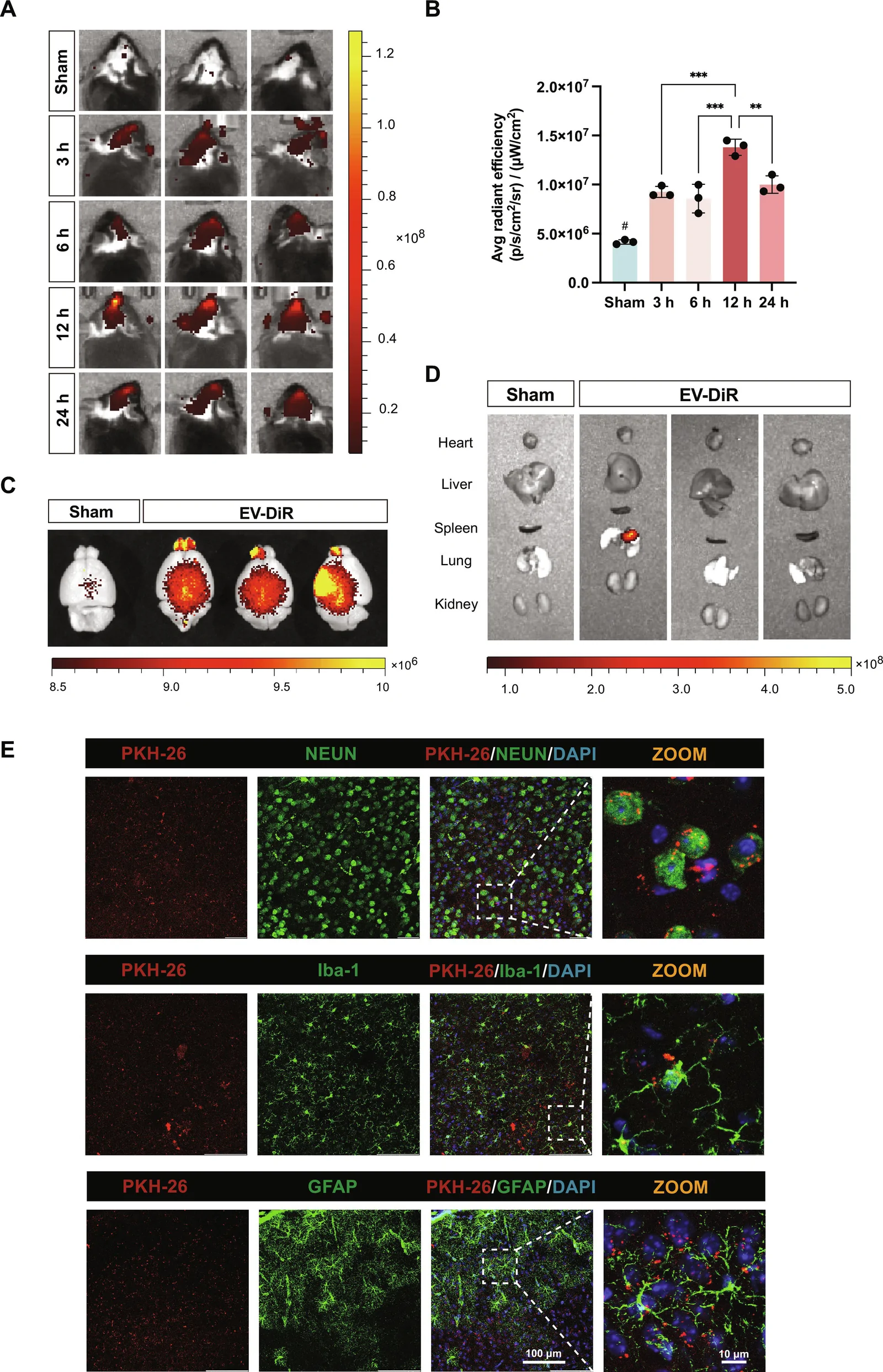
Intranasal administration enables EV delivery to the ischemic brain and uptake by neural cells. (A) In vivo fluorescence imaging of mice following intranasal administration of DiR-labeled EVs at 3, 6, 12, and 24 h. (B) Quantification of average radiant efficiency in the brain region at the indicated time points. (C) Ex vivo fluorescence imaging of brain tissues 24 h after intranasal administration of DiR-labeled EVs. (D) Ex vivo fluorescence imaging of major organs (heart, liver, spleen, lung, and kidney) 24 h after intranasal administration of DiR-labeled EVs. Quantitative ROI analysis of fluorescence intensity in major organs is shown in Fig. S2. (E) Immunofluorescence staining of brain sections from tMCAO mice 24 h after intranasal administration of PKH26-labeled EVs. PKH26-labeled EVs (red) colocalized with neurons (NeuN), microglia (Iba1), and astrocytes (GFAP) in the ischemic region. Scale bars, 100 μm or 10 μm. Data are presented as mean ± SD (*N* = 3 per group). ^#^*P* < 0.05 versus sham; **P < 0.01, ***P < 0.001 for comparisons among indicated time points in (B).

To further assess tissue distribution, ex vivo imaging of the brain and major organs was performed 24 h after administration. The EV-DiR group exhibited significantly increased fluorescence signal in the brain compared with the sham group, with stronger fluorescence observed in the ischemic hemisphere (Fig. 3C). Quantitative region of interest (ROI) analysis confirmed that the average radiant efficiency in the brain was significantly higher in the EV-DiR group than in the sham group, whereas no significant differences were observed in the heart, liver, spleen, lung, or kidney after correction for multiple comparisons (Fig. S2). Although a relatively high mean fluorescence signal was observed in the lungs of the EV-DiR group, substantial interindividual variability was evident, and the difference did not reach statistical significance (Fig. 3D and Fig. S2).

We next investigated the cellular localization of intranasally administered EVs in the ischemic brain. PKH26-labeled EVs were administered intranasally to tMCAO mice. Immunofluorescence staining performed 24 h after administration showed colocalization of PKH26 fluorescence with neurons (NeuN-positive), microglia (Iba1-positive), and astrocytes (GFAP-positive) in the ischemic region (Fig. 3E). Among these cell types, PKH26 fluorescence was more frequently observed in NeuN-positive neurons, suggesting that neurons are a major population of cells internalizing intranasally delivered EVs.

Taken together, these results demonstrate that intranasally administered EVs can reach the ischemic brain and be internalized by multiple neural cell types, with neurons representing a major cell population showing EV uptake.

### Intranasally administered EV@mNetrin-1 enhances Netrin-1 expression in the ischemic brain

To determine whether intranasal administration of EV@mNetrin-1 enhances Netrin-1 expression in the ischemic brain, tMCAO mice were treated with PBS, EV, or EV@mNetrin-1, with sham-operated mice serving as controls. Ischemic brain tissues were collected 3 d after treatment for analysis. Western blot analysis demonstrated significantly increased Netrin-1 protein expression in the EV@mNetrin-1 group compared with the PBS and EV groups, whereas no significant difference was observed between the PBS and EV groups (Fig. 4A and B).

**Fig. 4.**
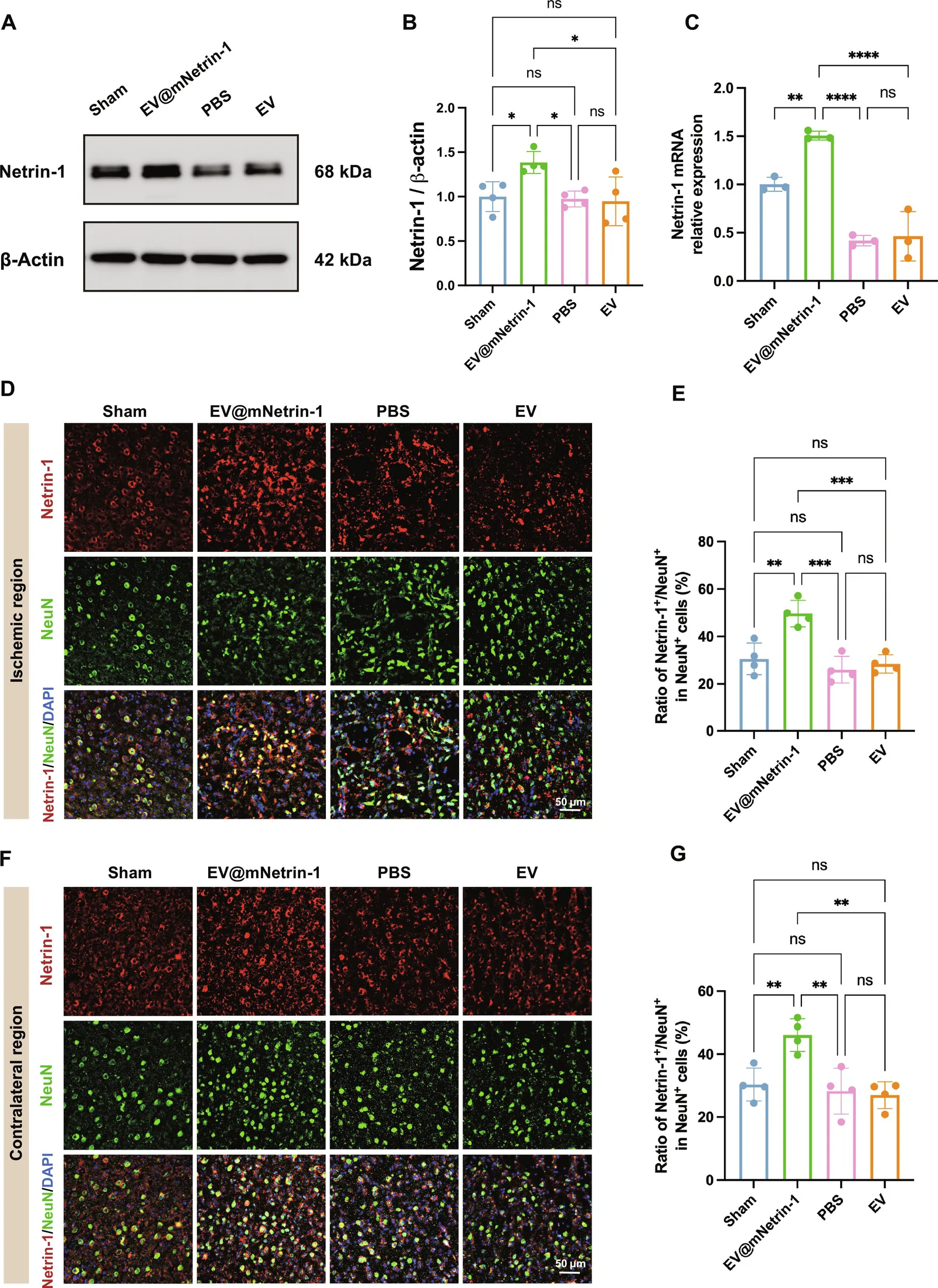
Intranasal administration of EV@mNetrin-1 enhances Netrin-1 expression in the ischemic brain and neurons. (A) Western blot analysis of Netrin-1 protein expression in the ischemic hemisphere at 3 d after tMCAO in sham, PBS, EV, and EV@mNetrin-1 groups. *N* = 4 per group. (B) Quantification of Netrin-1 protein expression shown in (A). (C) qPCR analysis of Netrin-1 mRNA expression in the ischemic hemisphere. *N* = 3 per group. (D and E) Representative immunofluorescence images showing Netrin-1 (red) and NeuN (green) staining and quantitative analysis of the percentage of Netrin-1^+^/NeuN^+^ cells among NeuN^+^ cells in the ischemic hemisphere. *N* = 4 per group. (F and G) Representative immunofluorescence images showing Netrin-1 (red) and NeuN (green) staining and quantitative analysis of the percentage of Netrin-1^+^/NeuN^+^ cells among NeuN^+^ cells in the contralateral hemisphere. *N* = 4 per group. Scale bars, 50 μm. Data are presented as mean ± SD. *P < 0.05; **P < 0.01; ***P < 0.001; ****P < 0.0001; ns, not significant.

Similarly, qPCR analysis showed significantly elevated Netrin-1 mRNA levels in the EV@mNetrin-1 group compared with the sham, PBS, and EV groups (Fig. 4C). In contrast, both the PBS and EV groups exhibited reduced Netrin-1 mRNA expression relative to the sham group, with no significant difference between the 2 groups.

Given that intranasally administered EVs were preferentially internalized by neurons, we next examined whether the increased Netrin-1 expression was localized to neurons. Immunofluorescence staining demonstrated colocalization of Netrin-1 fluorescence (red) with NeuN-positive neurons (green) in both the ischemic hemisphere (Fig. 4D and E) and the contralateral hemisphere (Fig. 4F and G). Quantitative analysis showed that the percentage of Netrin-1^+^/NeuN^+^ cells among NeuN^+^ cells was significantly increased in the EV@mNetrin-1 group compared with all other groups in both regions.

Taken together, these results demonstrate that intranasal administration of EV@mNetrin-1 enhances Netrin-1 expression in the brain, particularly in neurons, indicating successful in vivo delivery and expression of Netrin-1 modRNA.

### EV@mNetrin-1 reduces ischemic brain injury and improves neurological function in tMCAO mice

To determine whether EV@mNetrin-1 exerts neuroprotective effects in vivo, tMCAO mice were treated with PBS, EV, or EV@mNetrin-1, with sham-operated mice serving as controls. The experimental design is illustrated in Fig. 5A. Neurological function and histological analyses were performed 3 d after treatment.

**Fig. 5.**
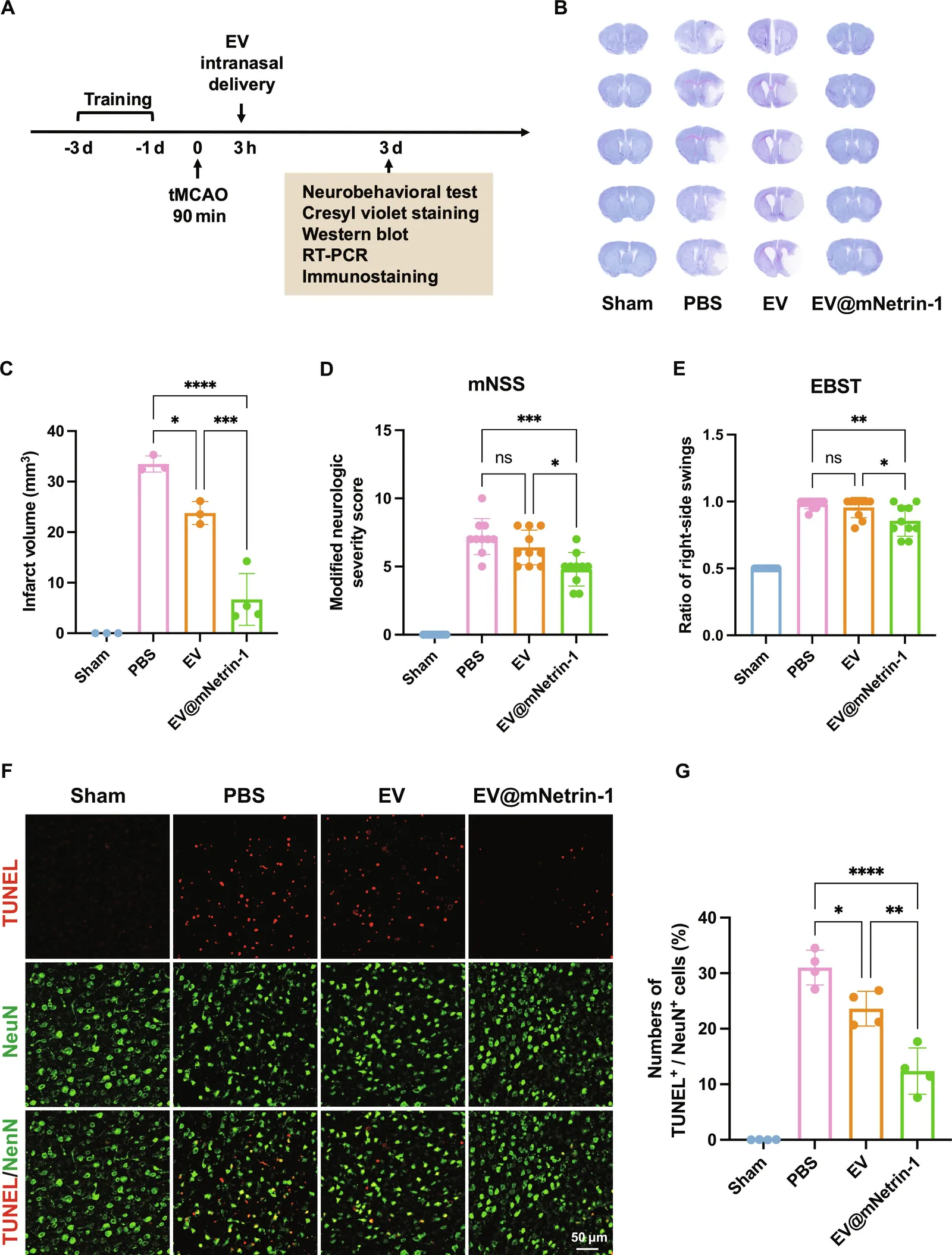
EV@mNetrin-1 reduces infarct volume, improves neurological function, and attenuates neuronal apoptosis in tMCAO mice. (A) Schematic diagram of the experimental design. (B) Representative cresyl violet staining images of brain sections at 3 d after tMCAO. (C) Quantification of infarct volume based on cresyl violet staining. *N* = 4 per group. (D) Neurological function assessed by mNSS. *N* = 10 per group. (E) Neurological function assessed by EBST. *N* = 10 per group. (F) Representative immunofluorescence images of TUNEL/NeuN costaining in brain sections. (G) Quantification of TUNEL-positive neurons. *N* = 4 per group. Scale bars, 50 μm. Data are presented as mean ± SD. *P < 0.05; **P < 0.01; ***P < 0.001; ns, not significant.

Cresyl violet staining showed that infarct volume was significantly reduced in both the EV and EV@mNetrin-1 groups compared with the PBS group (Fig. 5B and C). Moreover, EV@mNetrin-1 treatment further decreased infarct volume compared with EV treatment. Consistent with the reduced infarct volume, the EV@mNetrin-1 group exhibited significantly improved neurological function, reflected by lower mNSS scores and improved EBST performance compared with the PBS and EV groups (Fig. 5D and E). Although the EV group showed a trend toward functional improvement relative to the PBS group, the difference was not statistically significant.

We next examined whether the neuroprotective effects of EV@mNetrin-1 were associated with reduced neuronal apoptosis in the ischemic brain. TUNEL/NeuN costaining showed that the number of TUNEL-positive neurons was significantly reduced in both the EV and EV@mNetrin-1 groups compared with the PBS group (Fig. 5F and G). Moreover, EV@mNetrin-1 treatment further decreased neuronal apoptosis relative to EV treatment.

Taken together, these findings demonstrate that EV@mNetrin-1 exerts neuroprotective effects in tMCAO mice, characterized by reduced infarct volume, improved neurological function, and decreased neuronal apoptosis.

### EV@mNetrin-1 promotes PI3K/AKT signaling and attenuates neuronal apoptosis in the ischemic brain

To further investigate the signaling mechanisms associated with the neuroprotective effects of EV@mNetrin-1, the PI3K/AKT pathway and apoptosis-related proteins were evaluated in the ischemic brain 3 d after treatment. Western blot analysis was performed to assess p-PI3K, PI3K, p-AKT, AKT, BCL2, BAX, and cleaved caspase-3 expression (Fig. 6A).

**Fig. 6.**
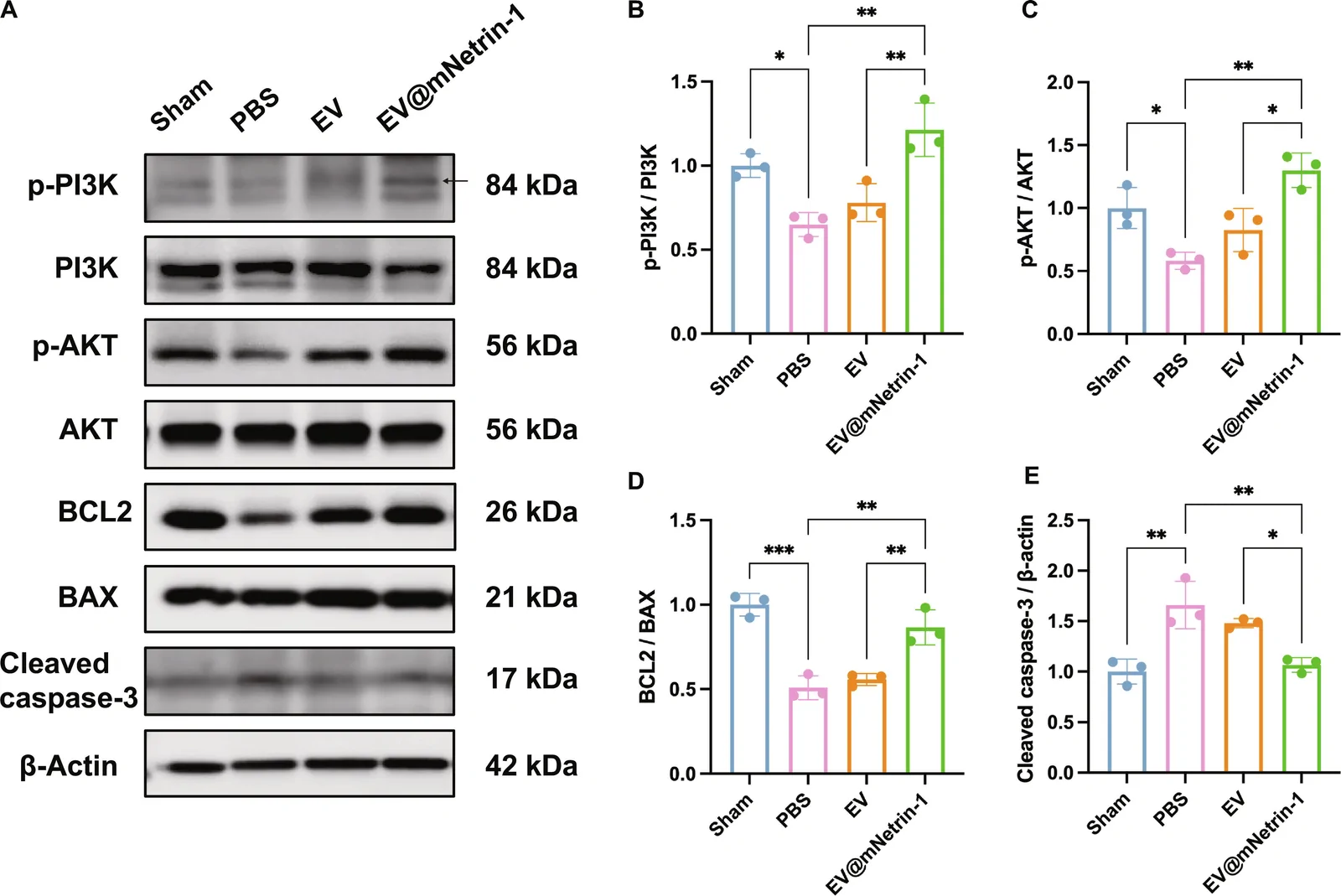
EV@mNetrin-1 enhances PI3K/AKT signaling and regulates apoptosis-related proteins in the ischemic brain. (A) Western blot analysis of p-PI3K, PI3K, p-AKT, AKT, BCL2, BAX, and cleaved caspase-3 expression in ischemic brain tissues at 3 d after tMCAO in PBS-, EV-, and EV@mNetrin-1-treated mice. The arrows indicate the specific p-PI3K band (~84 kDa) used for densitometric analysis. (B) Quantification of the p-PI3K/PI3K ratio. (C) Quantification of the p-AKT/AKT ratio. (D) Quantification of the BCL2/BAX ratio. (E) Quantification of cleaved caspase-3 protein expression. *N* = 3 per group. Data are presented as mean ± SD. *P < 0.05, **P < 0.01, ***P < 0.001.

Quantitative analysis revealed that the p-PI3K/PI3K ratio was significantly increased in the EV@mNetrin-1 group compared with the PBS and EV groups (Fig. 6B). Similarly, the p-AKT/AKT ratio was significantly elevated in the EV@mNetrin-1 group relative to the PBS and EV groups (Fig. 6C). Although the EV group showed a trend toward increased PI3K/AKT signaling compared with the PBS group, the differences were not statistically significant.

We next examined whether PI3K/AKT activation was accompanied by changes in apoptosis-related proteins. The BCL2/BAX ratio was significantly increased, whereas cleaved caspase-3 expression was significantly reduced in the EV@mNetrin-1 group compared with the PBS and EV groups (Fig. 6D and E). In contrast, EV treatment alone did not significantly alter these apoptosis-related proteins compared with PBS treatment.

Taken together, these findings suggest that the neuroprotective effects of EV@mNetrin-1 are associated with activation of the PI3K/AKT signaling pathway and reduced neuronal apoptosis in the ischemic brain.

### DCC is involved in EV@mNetrin-1-induced PI3K/AKT activation and attenuation of neuronal apoptosis

Given the predominant expression of DCC in neurons and its role in neuronal survival signaling, we next investigated whether DCC was involved in the neuroprotective effects of EV@mNetrin-1. Primary neurons were transfected with DCC-specific siRNA to knock down DCC expression prior to OGD/R treatment.

DCC knockdown efficiency was first confirmed. qPCR analysis showed that DCC mRNA expression was reduced to approximately 29.36% of the control level (Fig. 7A). Correspondingly, Western blot analysis demonstrated that DCC protein expression was reduced to approximately 40% of the control level (Fig. 7B and C), confirming effective DCC knockdown in primary neurons.

**Fig. 7.**
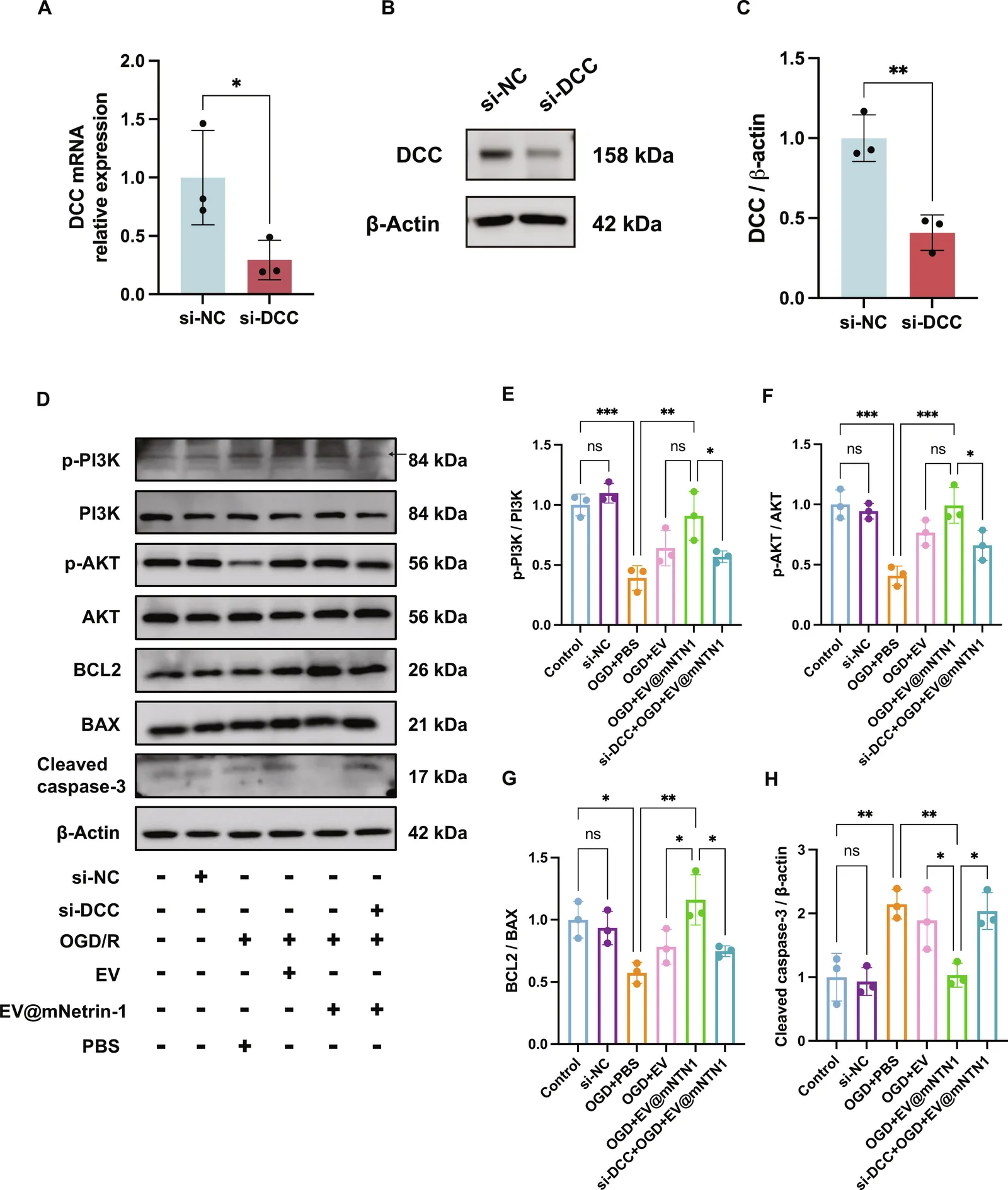
DCC is involved in EV@mNetrin-1-induced activation of PI3K/AKT signaling and attenuation of neuronal apoptosis. (A) qPCR analysis of DCC mRNA expression in primary neurons following transfection with DCC-specific siRNA. (B and C) Western blot analysis and quantification of DCC protein expression after siRNA-mediated knockdown. (D) Western blot analysis of p-PI3K, PI3K, p-AKT, AKT, BCL2, BAX, and cleaved caspase-3 in primary neurons under the indicated treatment conditions. Primary neurons were subjected to OGD/R and treated with PBS, EVs, or EV@mNetrin-1, with or without DCC knockdown. Untreated and si-NC groups without OGD/R were included as controls. The arrows indicate the specific p-PI3K band (~84 kDa) used for densitometric analysis. (E) Quantification of the p-PI3K/PI3K ratio. (F) Quantification of the p-AKT/AKT ratio. (G) Quantification of the BCL2/BAX ratio. (H) Quantification of cleaved caspase-3 protein expression. *N* = 3 per group. Data are presented as mean ± SD. *P < 0.05; **P < 0.01; ***P < 0.001; ns, not significant. EV@mNTN1, EV@mNetrin-1.

To determine whether DCC knockdown affected EV@mNetrin-1-induced signaling changes, neurons were subjected to OGD/R and treated with PBS, EV, or EV@mNetrin-1 in the presence or absence of DCC knockdown. Representative Western blot images of PI3K/AKT signaling and apoptosis-related proteins in neurons collected 24 h after reoxygenation are shown in Fig. 7D. No significant differences were observed between the control and si-NC groups, indicating that siRNA transfection alone did not significantly affect these signaling pathways.

Following OGD/R, the p-PI3K/PI3K ratio was significantly increased in the EV@mNetrin-1 group compared with the PBS group, whereas the EV group showed a trend toward increased PI3K activation without statistical significance. Importantly, DCC knockdown significantly attenuated the increase in p-PI3K/PI3K induced by EV@mNetrin-1 treatment (Fig. 7E).

Consistently, the p-AKT/AKT ratio was significantly increased in both the EV and EV@mNetrin-1 groups compared with the PBS group, with a greater increase observed in the EV@mNetrin-1 group. DCC knockdown partially attenuated EV@mNetrin-1-induced AKT activation (Fig. 7F).

In parallel with these signaling changes, the BCL2/BAX ratio was significantly increased, whereas cleaved caspase-3 expression was significantly reduced in the EV@mNetrin-1 group compared with the PBS group (Fig. 7G and H). These apoptosis-related changes were partially reversed following DCC knockdown.

Taken together, these findings indicate that DCC is involved in EV@mNetrin-1-mediated activation of PI3K/AKT signaling and attenuation of neuronal apoptosis under ischemic conditions.

The schematic in Fig. 8 summarizes the proposed neuroprotective mechanism of EV@mNetrin-1 after acute ischemic stroke. Following intranasal administration, EV@mNetrin-1 is delivered to the ischemic brain and internalized by neurons, where Netrin-1 modRNA is translated into Netrin-1 protein. Increased Netrin-1 expression is associated with DCC-mediated activation of PI3K/AKT signaling, reduced neuronal apoptosis, and improved neurological outcomes.

**Fig. 8.**
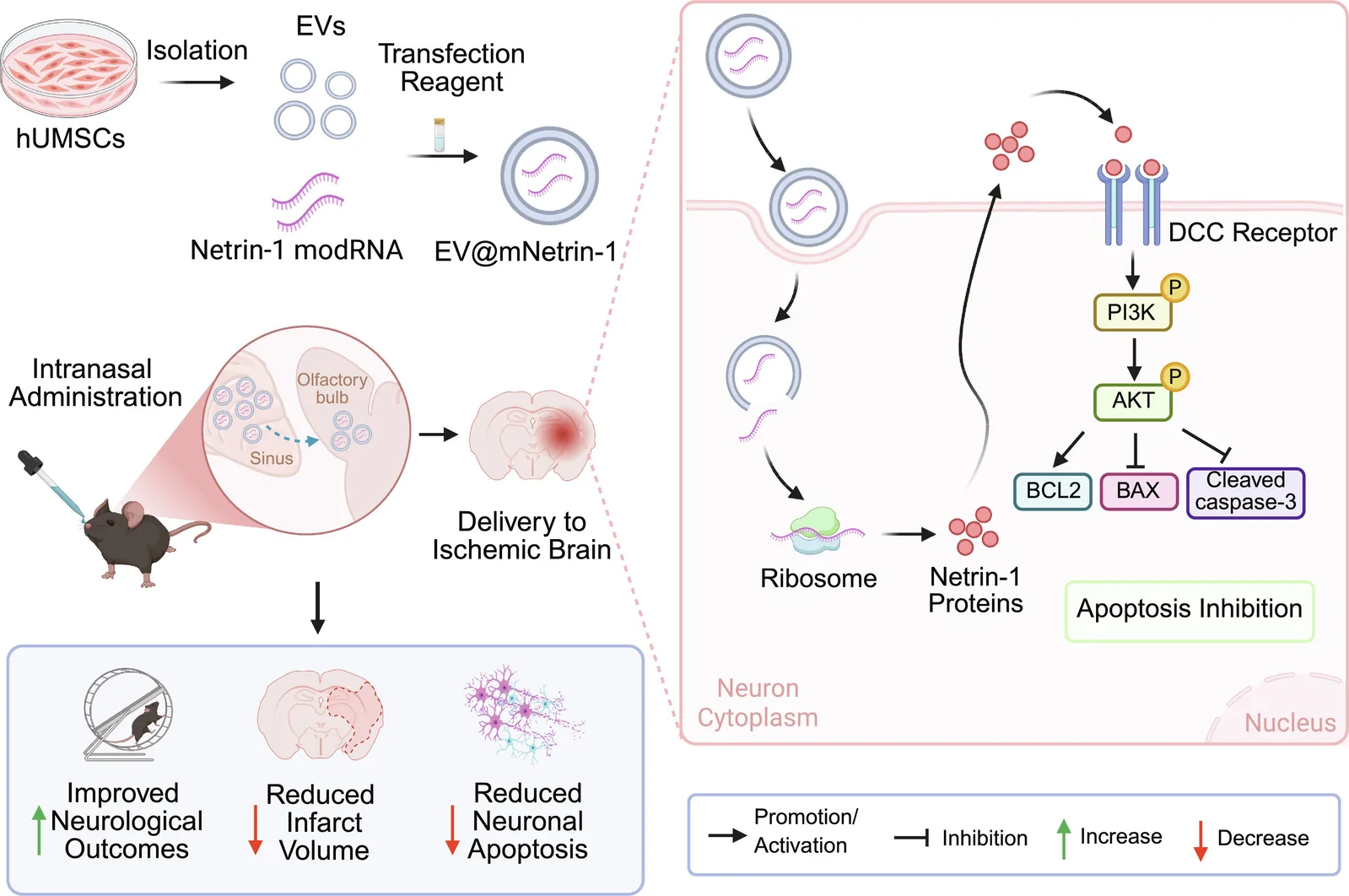
Schematic illustration of EV@mNetrin-1 preparation, intranasal delivery, and its proposed neuroprotective mechanism in acute ischemic stroke. Following intranasal administration, EV@mNetrin-1 is delivered to the ischemic brain and internalized by neurons, where Netrin-1 modRNA is translated into Netrin-1 protein. Increased Netrin-1 is associated with DCC-mediated activation of PI3K/AKT signaling, reduced neuronal apoptosis, and improved neurological outcomes.

## Discussion

In the present study, we engineered hUMSC-derived EVs loaded with Netrin-1 modRNA (EV@mNetrin-1) and demonstrated their therapeutic potential in ischemic stroke. In vitro, EV-mediated delivery enabled efficient intracellular transfer of Netrin-1 modRNA into primary neurons and subsequent translation into Netrin-1 protein. In vivo, intranasally administered EVs effectively reached the ischemic brain and showed abundant localization in neurons. Treatment with EV@mNetrin-1 significantly enhanced Netrin-1 expression in the ischemic brain, reduced neuronal apoptosis and infarct volume, and improved neurological outcomes in tMCAO mice. Mechanistically, our findings further suggest that the neuroprotective effects of EV@mNetrin-1 are mediated, at least in part, through DCC-dependent activation of PI3K/AKT signaling. Collectively, these findings highlight the therapeutic potential of EV@mNetrin-1 as a promising neuroprotective strategy for ischemic stroke.

mRNA therapy is a promising therapeutic strategy that delivers protein-coding information to enable intracellular synthesis of target proteins, thereby facilitating disease intervention [8,9,22–24]. It is characterized by transient yet efficient protein expression in target cells. Compared with DNA- or protein-based therapeutics, mRNA offers several advantages, including a favorable safety profile due to the lack of genomic integration and the ability to enable in situ protein production that more closely mimics physiological processes. In certain contexts, this may also result in more sustained protein availability than direct protein administration [16,25]. However, naked mRNA is limited by its negative charge and susceptibility to degradation, which impair cellular uptake and necessitate the use of delivery vectors. Gao et al. [26] used M2 microglia-targeting LNPs to deliver interleukin-10 (IL-10)-encoding mRNA, promoting microglial polarization from the proinflammatory M1 phenotype to the reparative M2 phenotype, thereby restoring BBB integrity and improving neurological outcomes after ischemic stroke. Emerging evidence further suggests that EVs may offer advantages over synthetic lipid-based carriers for mRNA delivery [14,27]. Compared with liposomal systems, EV-mediated mRNA delivery exhibits enhanced cellular uptake and deeper tissue penetration, likely due to improved mucus permeability [28]. In addition, IL-12 mRNA-loaded EVs achieved greater pulmonary accumulation and reduced off-target effects than liposomal counterparts in lung cancer models [29]. Collectively, these findings highlight the potential of EVs as an efficient and biocompatible delivery platform for modRNA, thereby providing a strong rationale for their application in the present study.

Cargo loading into EVs can be broadly classified into endogenous and exogenous strategies [30,31]. Endogenous loading, achieved through genetic engineering of donor cells, is often time-consuming and limited by low loading efficiency. In contrast, exogenous loading introduces cargo into purified EVs, offering greater flexibility and efficiency. In this study, we employed an exogenous approach using the Exo-Fect reagent to load Netrin-1 modRNA into hUMSC-derived EVs, thereby generating engineered EVs. Conventional methods such as electroporation, although widely used, may induce EV aggregation, membrane disruption, or loss of surface proteins, potentially compromising their biological function [32]. In comparison, Exo-Fect provides a relatively mild and practical approach for nucleic acid loading, which may better preserve EV membrane integrity and intrinsic biological activity. Consistently, our results showed that Exo-Fect-engineered EVs retained typical EV characteristics, including morphology, size distribution, and marker expression, while efficiently delivering Netrin-1 modRNA into neurons and significantly enhancing Netrin-1 expression.

Intranasal delivery represents a noninvasive route that can bypass the BBB, thereby providing a potential strategy for direct transport of therapeutic agents to the brain [17,33]. Following intranasal administration, EVs can access the brain parenchyma via multiple pathways, including the olfactory and trigeminal nerve routes, as well as vascular and glymphatic systems, enabling effective delivery to the central nervous system. In the present study, in vivo imaging of tMCAO mice receiving intranasally administered DiR-labeled EVs revealed a significant increase in brain-associated fluorescence as early as 3 h, peaking at 12 h and remaining elevated at 24 h, suggesting rapid brain entry and sustained presence of EVs. These findings are consistent with previous reports demonstrating efficient brain distribution of EVs following intranasal delivery. Ex vivo imaging further showed stronger EV-associated fluorescence in the ischemic hemisphere at 24 h post-administration. Quantitative analysis confirmed a significant increase in brain fluorescence, whereas no significant differences were observed in the heart, liver, spleen, lung, or kidney after correction for multiple comparisons. The stronger fluorescence observed in the ischemic hemisphere may be related to ischemia-induced alterations in BBB permeability and the local tissue microenvironment, potentially facilitating EV accumulation and cellular uptake at the lesion site. Although a relatively high mean fluorescence signal was observed in the lungs, substantial interindividual variability was evident and the difference did not reach statistical significance; this variability may partly reflect variable aspiration during intranasal administration. Immunofluorescence analysis further demonstrated that intranasally delivered EVs were internalized by multiple neural cell types, including neurons, microglia, and astrocytes, with EV-associated signals frequently detected in neurons. This cellular distribution pattern is in line with previous findings and suggests a potential neuronal uptake preference of EVs in the brain, which may contribute to their neuroprotective effects [18,21].

EV@mNetrin-1 further enhanced the therapeutic efficacy of native EVs. In the tMCAO model, intranasal administration of EV@mNetrin-1 resulted in a significant increase in Netrin-1 protein levels in the ischemic brain at 3 d post-treatment, accompanied by reduced neuronal apoptosis, improved neurological function, and decreased infarct volume compared with EV treatment alone. These findings suggest that EV@mNetrin-1 can be efficiently internalized by neural cells and promote Netrin-1 protein expression, thereby exerting enhanced neuroprotective effects during the early phase of ischemic stroke. Mechanistically, EV@mNetrin-1 treatment activated PI3K/AKT signaling in ischemic brain tissue, accompanied by an increased BCL2/BAX ratio and reduced cleaved caspase-3 expression, suggesting suppression of apoptosis-related signaling. Consistent results were observed in primary neurons subjected to OGD/R in vitro, where EV@mNetrin-1 induced similar molecular changes. Notably, knockdown of the DCC receptor using siRNA markedly attenuated these signaling alterations and the associated anti-apoptotic effects, suggesting that the neuroprotective actions of EV@mNetrin-1 are at least partially dependent on DCC-mediated signaling. It is worth noting that the biological functions of Netrin-1 extend beyond the regulation of neuronal apoptosis. Netrin-1 has also been implicated in the modulation of inflammation, maintenance of BBB integrity, angiogenesis, and myelination processes [4]. Therefore, EV@mNetrin-1 may exert multifaceted protective effects following ischemic stroke. Further studies are warranted to elucidate its broader roles within the neurovascular unit and to explore its potential for translational application.

From a clinical perspective, effective neuroprotective strategies for ischemic stroke remain limited, particularly due to reperfusion injury and the narrow therapeutic time window of current interventions. In this study, we combined intranasal administration with EV-based modRNA delivery to achieve noninvasive brain delivery of therapeutic molecules, which may complement existing reperfusion therapies. This approach shows translational potential, as intranasal delivery is clinically feasible and minimally invasive, EVs exhibit favorable biocompatibility and low immunogenicity, and modRNA enables transient, controllable protein expression without genomic integration. Overall, EV-mediated intranasal modRNA delivery represents a promising strategy for neurological disorders. Several limitations should be acknowledged. First, this study was conducted in a mouse model of ischemic stroke, and its translational relevance to human patients requires further validation. Second, the current study primarily focused on short-term therapeutic effects and neuronal apoptosis, while long-term efficacy and functional recovery remain to be determined. Third, the mechanistic investigation was mainly centered on DCC-mediated signaling, and the involvement of additional molecular pathways cannot be excluded. Future studies should further evaluate the multifaceted therapeutic effects of EV@mNetrin-1 in ischemic brain injury, elucidate its mechanisms within the neurovascular unit, and assess long-term safety and translational potential.

In this study, we engineered EVs carrying Netrin-1 modRNA and demonstrated their neuroprotective effects in ischemic stroke following intranasal administration. EV@mNetrin-1 enhanced Netrin-1 expression in the ischemic brain, reduced neuronal apoptosis and infarct volume, and improved neurological function, with protective effects at least partially mediated through DCC-dependent signaling. Collectively, these findings support intranasal delivery of EV@mNetrin-1 as a promising therapeutic strategy for neuroprotection after ischemic stroke.

## Data Availability

All data supporting the findings of this study are available from the corresponding author upon reasonable request.
