# Supplementary material for: Intranasal Netrin-1 modRNA-Loaded Extracellular Vesicles Reduce Neuronal Apoptosis after Ischemic Stroke
Source: Biomater Res. 2026 Oct 6;30:0423. doi: 10.34133/bmr.0423 (PMC13639143; doi:10.34133/bmr.0423)
Supplement: Supplementary file 1 [file bmr.0423.f1.docx]

**Supplementary Materials**

**Intranasal Netrin-1 modRNA-Loaded Extracellular Vesicles Reduce Neuronal Apoptosis After Ischemic Stroke**

Shanshan Cao^1^, Anmin Ren^2^, Yanqin Geng^3^, Hanlai Li^3^, Yandi Qiao^1,4^, Kangjie Fan^1,4^, Yaohui Tang^3^, Peng Wang^5*^, Qiang Li^1*^

**Affiliations**

^1^Department of Neurology, Gongli Hospital of Shanghai Pudong New Area, Shanghai 200135, China.

^2^Department of Cardiology, Shanghai General Hospital, Shanghai Jiao Tong University of Medicine, Shanghai 200080, China.

^3^School of Biomedical Engineering, Shanghai Jiao Tong University, Shanghai 200030, China.

^4^School of Gongli Hospital Medical Technology, University of Shanghai for Science and Technology, Shanghai 200093, China.

^5^Anti-Aging Center, Gongli Hospital of Shanghai Pudong New Area, Shanghai 200135, China.

^*^Address correspondence to: Peng Wang; <wp113000@163.com> and Qiang Li; <lqsmmu@126.com>

**Fig. S1. Additional characterization of EV@mNetrin-1.** (A) Quantitative analysis of modal particle diameter of EVs and EV@mNetrin-1 determined by nanoparticle tracking analysis (NTA). (B) Quantitative analysis of mean particle diameter of EVs and EV@mNetrin-1 determined by NTA. (C) Western blot analysis of Calnexin expression in hUMSCs, EVs, and EV@mNetrin-1. (D) Quantification of the encapsulation efficiency (EE) of Netrin-1 modRNA in EV@mNetrin-1. Data are presented as mean ± SD. **p* < 0.05, ns, not significant.

**Fig. S2. Quantitative analysis of ex vivo biodistribution of DiR-labeled EVs in major organs following intranasal administration.** Average radiant efficiency was quantified by ROI analysis in the brain, heart, liver, spleen, lung, and kidney 24 h after intranasal administration. Data are presented as mean ± SD (*N* = 3 per group). Statistical comparisons between the Sham and EV-DiR groups for each organ were performed using multiple unpaired two-tailed Welch’s t-tests with the Holm-Šídák correction for multiple comparisons. Adjusted *p* values are reported; **p* < 0.05; ns, not significant.

**Table S1. Primer sequences of RT-qPCR**

| Gene | Forward primer (5′-3′) | Reverse primer (5′-3′) |
| --- | --- | --- |
| Netrin-1 | GCGCGACTCCTATTACTATG | CTTGCAACGGTCGCATTCAG |
| DCC | CCACCTTCCAACTGGTACACAG | GAGAGGAGGTGTCCAACTCATG |
| β-actin | GGCTGTATTCCCCTCCATCG | CCAGTTGGTAACAATGCCATGT |
